# Iron-responsive ZNF185 overexpression drives mitochondrial fission and endoplasmic reticulum stress via cytoskeletal remodeling in granulosa cells

**DOI:** 10.1038/s41420-025-02719-y

**Published:** 2025-08-28

**Authors:** Zhaoyue Huang, Yang You, Qi Qiu, Nan Dong, Xinye Hu, Meihong Cai, Yaoqiu Wu, Chunwei Cao, Qingxue Zhang

**Affiliations:** 1 Reproductive Medicine Center, Sun Yat-sen Memorial Hospital, Sun Yat-sen University, Guangzhou, Guangdong China; 2 Shenzhen Maternity and Child Healthcare Hospital, Women and Children’s Medical Center, Southern Medical University, Shenzhen, Guangdong China; 3 Guangdong Provincial Key Laboratory of Malignant Tumor Epigenetics and Gene Regulation, Guangdong-Hong Kong Joint Laboratory for RNA Medicine, Medical Research Center, Sun Yat-sen Memorial Hospital, Sun Yat-sen University, Guangzhou, China; 4 Guangzhou Laboratory, Guangzhou, China

**Keywords:** Endocrine reproductive disorders, Reproductive disorders

## Abstract

Ovarian endometrioma (OMA), an estrogen-dependent gynecological disorder, is characterized by the presence of abundant free iron resulting from recurrent hemorrhage of endometrial cells within the cyst, which adversely affects ovarian function. However, the underlying mechanisms through which iron overload impairs ovarian function remain unclear. In this study, we stimulated KGN cells with ferric ammonium citrate (FAC) in vitro and observed dose-dependent significant alterations, including decreased mitochondrial membrane potential, increased reactive oxygen species (ROS), decreased cell viability, and elevated apoptosis rates. RNA sequencing analysis of iron-overloaded KGN cells demonstrated significant upregulation of *ZNF185* expression across multiple concentration gradients and treatment durations. *ZNF185* overexpression was found to disrupt F-actin dynamics, triggering a cascade of cellular events including Drp1-mediated mitochondrial hyperfission, endoplasmic reticulum stress, and cytochrome C release, ultimately leading to granulosa cell apoptosis. Importantly, knockdown of *ZNF185* was shown to preserve cytoskeletal integrity and attenuate apoptotic responses under conditions of iron overload. Our findings demonstrated that *ZNF185* served as a novel iron-responsive regulator involved in iron overload-induced granulosa cell apoptosis. These results might provide potential therapeutic strategies for ovarian fertility preservation in OMA patients.

## Introduction

Ovarian endometrioma (OMA) is an estrogen-dependent gynecological disorder characterized by the invasion of endometrial tissue into the ovary, leading to cyclic hemorrhage and the formation of ectopic cysts [1, 2]. Clinical studies have demonstrated that ovarian endometrioma is associated with impaired ovarian response and diminished reserve function, manifesting as reduced oocyte yield, decreased antral follicle count (AFC), lower anti-Müllerian hormone (AMH) levels, and compromised embryo implantation rates [3, 4]. Ovarian endometrioma is characterized by cysts containing high concentrations of free iron, resulting from recurrent hemorrhage from ectopic endometrial cells during menstrual cycles [5, 6]. Evidence has indicated that toxic components within ovarian endometrioma, including iron, reactive oxygen species (ROS), nitric oxide, and inflammatory cytokines, exerted detrimental effects on adjacent ovarian tissues. These pathological changes led to impaired follicular maturation and reduced follicular density [7–9].

Iron is an indispensable micronutrient in humans, serving as a critical cofactor in fundamental biological processes such as ATP generation, oxygen transport, and DNA synthesis. Each of those is essential for cellular metabolism, tissue homeostasis, and overall growth and development [10]. However, excess intracellular iron can participate in the Fenton reaction with hydrogen peroxide, generating excessive ROS and promoting intracellular lipid peroxidation. This leads to enhanced cellular oxidative stress and inflammatory responses, ultimately inducing cell death and organ dysfunction [11]. For instance, iron overload in the myocardium can lead to heart failure and arrhythmias, while chronic hepatic iron overload may progress to fibrosis, subsequently developing into cirrhosis and even hepatocellular carcinoma. Endocrine organs are particularly vulnerable to iron overload, potentially manifesting as diabetes mellitus, hypothyroidism, or hypogonadism in young patients with β-thalassemia major [12, 13].

Within the ovarian microenvironment, iron overload has been shown to induce granulosa cell apoptosis, leading to follicular atresia and compromised oocyte development [14, 15]. Our prior research revealed that iron overload disrupts estrogen biosynthesis via the HIF-1α/FSHR/CYP19A1 signaling axis, consequently impairing follicular development and maturation [16]. However, the precise molecular mechanisms governing iron overload-mediated ovarian dysfunction remain poorly characterized, warranting further investigation.

To investigate the mechanisms underlying iron overload-associated ovarian dysfunction, we established an in vitro granulosa cell model based on the follicular fluid iron concentrations observed in patients with ovarian endometrioma. RNA sequencing identified *ZNF185* as a novel iron-responsive regulator. ZNF185 contains a C-terminal Lin-11, Isl-1, and Mec-3 (LIM)-type zinc finger domain that mediates protein-protein interactions [17], as well as an N-terminal actin-targeting domain (ATD) that co-localizes with F-actin. Our study demonstrated that iron overload upregulated *ZNF185* expression, triggering F-actin remodeling and subsequent Drp1 recruitment to promote mitochondrial fission. The cascade led to mitochondrial fragmentation, ROS accumulation, endoplasmic reticulum stress, and ultimately granulosa cell apoptosis, resulting in diminished ovarian reserve and impaired oocyte development.

In summary, we identify *ZNF185* as a novel iron-responsive factor that mediates iron overload-induced ovarian dysfunction. These findings may provide therapeutic insights for fertility preservation in patients with ovarian endometrioma.

## Results

### Ovarian reserve function was reduced in ovarian endometrioma patients

**Fig. 1:**
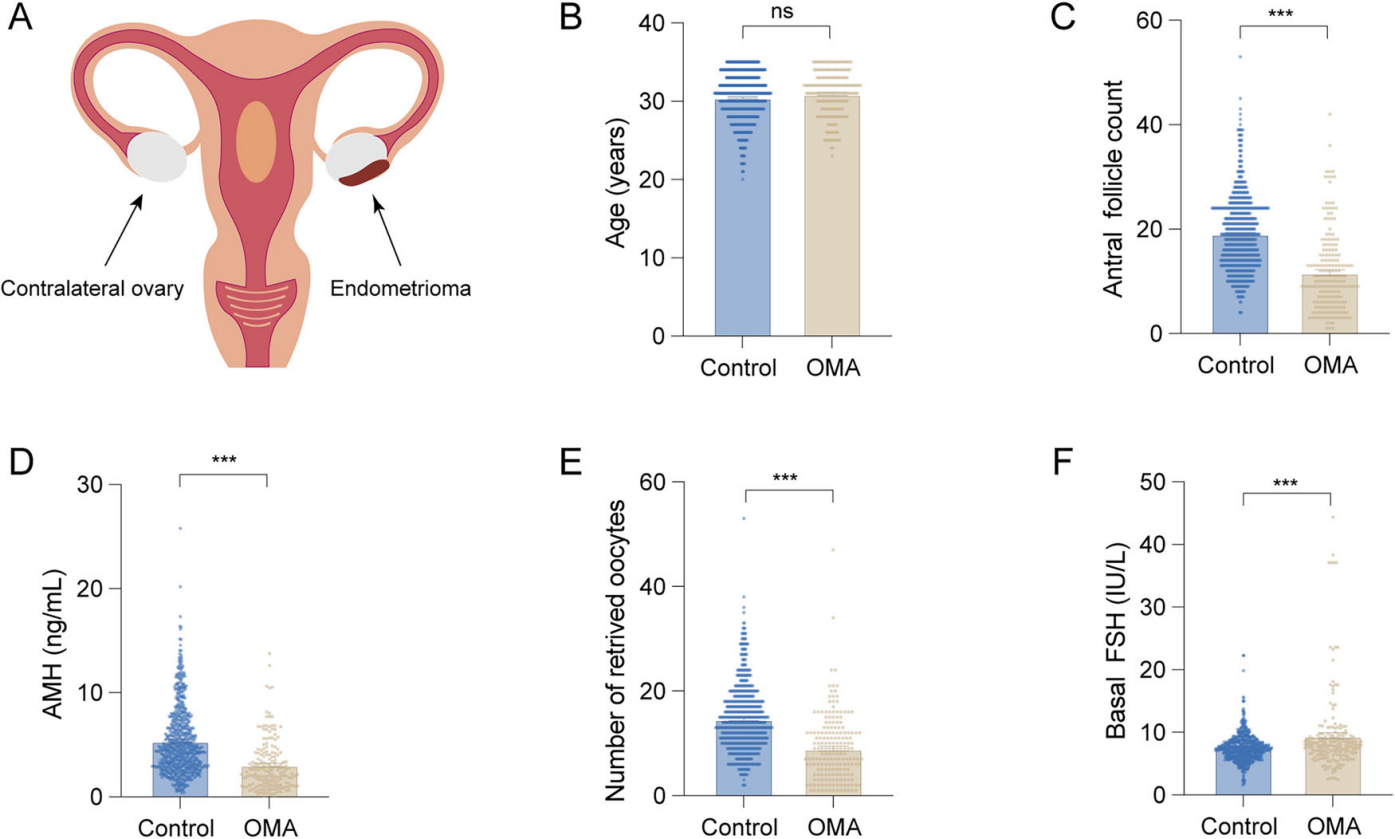
Age and ovarian reserve function of patients in the control group and ovarian endometrioma (OMA) group. **A** Schematic diagram of endometrioma. **B** Comparison of age between the control group (*n* = 712) and the OMA group (*n* = 187). **C**–**F** Comparison of ovarian reserve function between the two groups. Statistical analysis was performed using Student’s *t*-test. Data was shown as mean ± S.E.M. *P*-values were reported using GraphPad: not significant (ns), *P* > 0.05; *P < 0.05; **P < 0.01; ***P < 0.001.

This study included 187 patients with ovarian endometrioma (Fig. 1A) and 712 control patients with male factor infertility. By comparing the ovarian reserve function indicators between the two groups, including antral follicle count (AFC), serum anti-Müllerian hormone (AMH) levels, basal follicle-stimulating hormone (bFSH) levels, and the number of retrieved oocytes, we aimed to explore the differences between the two groups.

The results showed that there was no significant statistical difference in age between the control group and the ovarian endometrioma group (*P* = 0.053) (Fig. 1B). The ovarian endometrioma showed significant lower AFC (19.11 ± 7.10 vs. 11.69 ± 7.59, *P* < 0.001), reduced serum AMH levels (5.37 ± 3.27 vs. 3.08 ± 2.39 ng/mL, *P* < 0.001), and diminished number of retrieved oocytes (14.60 ± 6.29 vs. 8.99 ± 6.36, *P* < 0.001) compared to the control group (Fig. 1C–E). However, the ovarian endometrioma group had higher bFSH levels compared to the control group (7.51 ± 2.15 vs. 9.44 ± 6.50 IU/L, *P* < 0.001) (Fig. 1F). Our results indicated that ovarian endometrioma can impair the ovarian reserve function in affected patients.

### Iron overload affected the apoptosis, viability, and estrogen secretion of granulosa cells

**Fig. 2:**
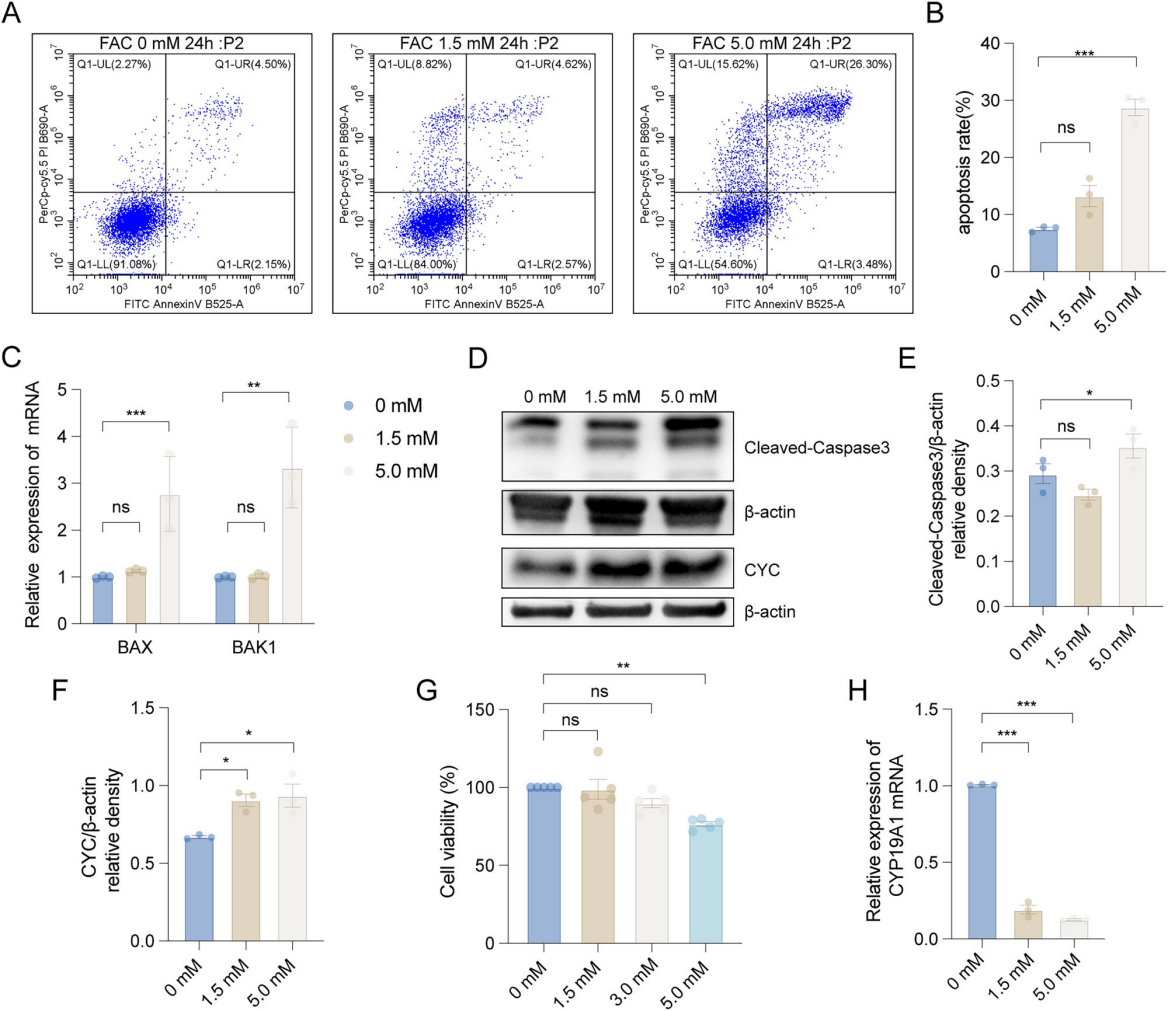
Effects of iron overload on apoptosis, viability, and estrogen secretion in KGN cells. **A**, **B** The apoptosis rate of KGN cells under various FAC concentrations for 24 h (*n* = 3). **C** Respective pro-apoptotic genes *BAX* and *BAK1* mRNA expression level was assessed by qPCR assay (*n* = 3). **D**–**F** Comparison of cytochrome C and cleaved Caspase-3 in KGN with FAC-treated and the control group (*n* = 3). **G** Histogram illustrated the cell viability of KGN decrease with the increase of FAC concentration (*n* = 5). **H** Estrogen secretion genes *CYP19A1* mRNA expression level was assessed by qPCR assay (*n* = 3). Statistical analysis was performed using one-way analysis of variance followed by the Bonferroni test. Data was shown as mean ± S.E.M. *P*-values were reported using GraphPad: not significant (ns), *P* > 0.05; *P < 0.05; **P < 0.01; ***P < 0.001.

To investigate the pathophysiological impact of iron overload on granulosa cell function, we established an in vitro model using the human granulosa-like tumor cell line (KGN). Cells were subjected to an iron challenge of 1.5 mM/24 h and 5.0 mM/24 h ferric ammonium citrate (FAC). Quantitative analysis revealed a dose-dependent increase in apoptosis, with apoptotic rates escalating from 7.50% in controls to 13.25% (1.5 mM FAC) and 28.76% (5.0 mM FAC) (*P* < 0.001, ANOVA) (Fig. 2A, B). Next, we measured the mRNA expression levels of the pro-apoptotic genes *BAX* and *BAK1* in KGN cells under the iron-overload conditions. The results indicated that FAC (5.0 mM) significantly induced the mRNA expression of *BAX* and *BAK1* (Fig. 2C). Moreover, the protein expression levels of the apoptosis-related proteins, cleaved Caspase-3 and cytochrome C (CYC), also increased along with the FAC concentration (Fig. 2D–F). These results confirmed that treatment with FAC in vitro can induce significant apoptosis in KGN cells.

Furthermore, we also investigated the effect of FAC stimulation for 24 h on the viability of KGN cells. The results showed that as the concentration of FAC increased, the viability of KGN cells decreased (Fig. 2G). To explore whether iron overload would affect the generation of estrogen in granulosa cells, we measured the mRNA levels of *CYP19A1* in the KGN cell line. The results showed that iron overload led to a significant decrease in the mRNA levels of *CYP19A1* in KGN cells (Fig. 2H). Collectively, these findings substantiated that excessive iron stimulation in vitro induced apoptosis, inhibited viability, and decreased the estrogen generation in KGN cells, which were consistent with our findings in granulosa cells from the follicular fluid of ovarian endometrioma patients.

### Iron overload-induced *ZNF185* expression in granulosa cells, disrupting F-actin cytoskeleton and triggering apoptosis

**Fig. 3:**
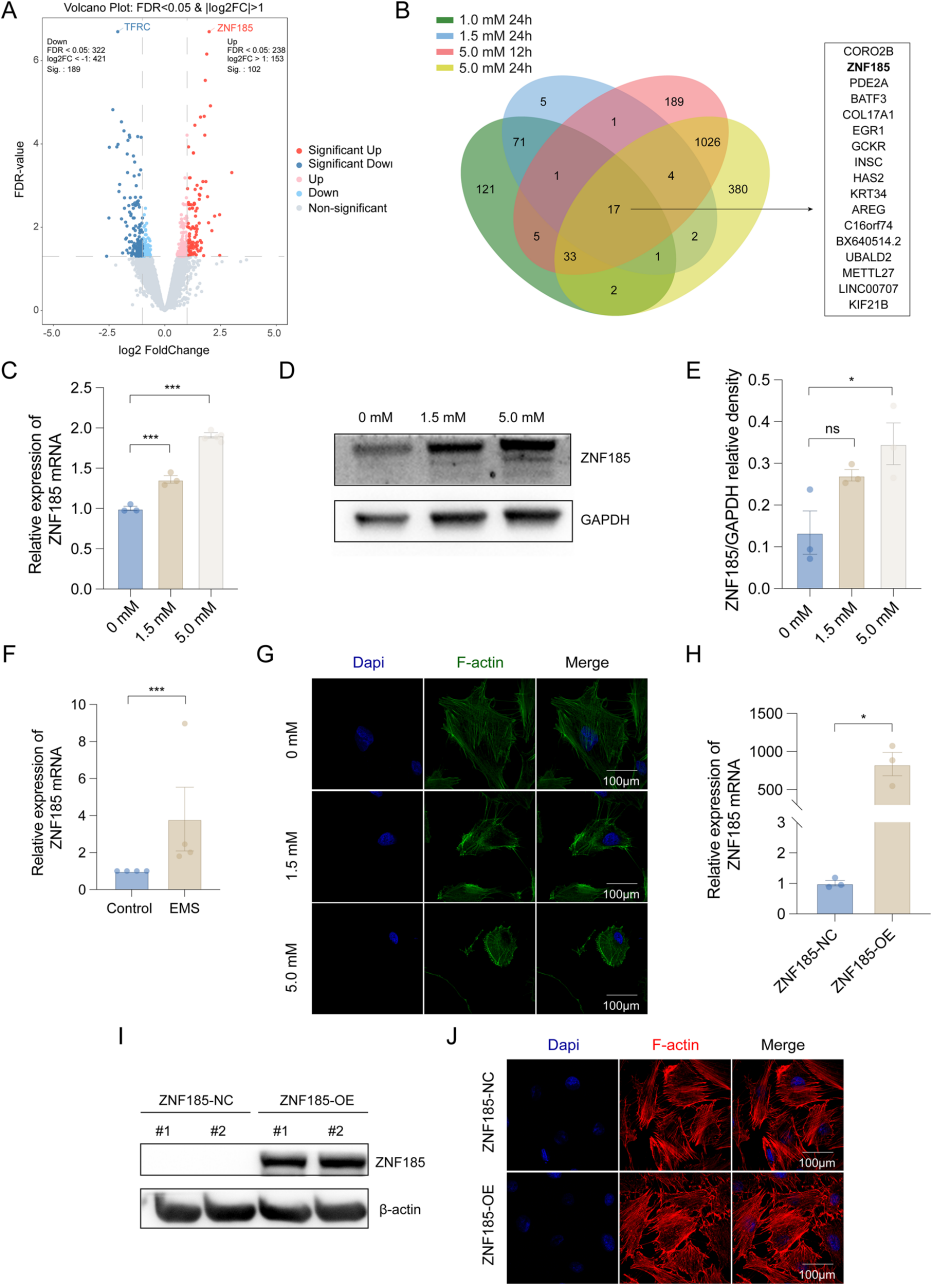
Correlation between *ZNF185* and iron overload-induced apoptosis in KGN cells. **A** Volcano plot of differentially expressed mRNAs (Foldchange ≥2, *P* < 0.05). **B** Venn diagram illustrating the overlap of upregulated genes identified across RNA sequencing groups of 1.0 mM/24 h, 1.5 mM/24 h, 5.0 mM/12 h, and 5.0 mM/24 h. **C** Real-time PCR validation of *ZNF185* expression in an iron overload circumstance (*n* = 3). **D**, **E** Comparison of ZNF185 in KGN with FAC-treated and the control group (*n* = 3). **F** The mRNA expression level of *ZNF185* in GCs that were isolated from follicle fluids of ovarian endometrioma (*n* = 4) and contralateral healthy ovary (*n* = 4). **G** Iron overload-induced disruption of the cytoskeleton F-actin of KGN cells. **H**, **I** Analysis of ZNF185 overexpression in stable KGN cell line by real-time PCR validation and Western Blots. **J** Morphology of cytoskeleton F-actin in stable KGN cell lines was exhibited. Statistical analysis was performed using Student’s *t*-test (two groups) and one-way analysis of variance followed by the Bonferroni test (more than two groups). Data was shown as mean ± S.E.M. *P*-values were reported using GraphPad: not significant (ns), *P* > 0.05; *P < 0.05; **P < 0.01; ***P < 0.001. Bar = 100 μm.

To delineate the molecular mechanisms underlying iron overload-induced apoptosis in KGN cells, we performed transcriptome profiling. RNA was extracted from cells exposed to ascending concentrations of ferric ammonium citrate (FAC; 1.0 mM, 1.5 mM, and 5.0 mM) and at two time points (12 h and 24 h for 1.5 mM and 5.0 mM) for high-throughput sequencing analysis (Table S1). RNA sequencing revealed marked upregulation of *ZNF185*, which was among the top differentially expressed genes in the 1.0 mM/24 h and 1.5 mM/24 h treatment groups (Fig. 3A, B), suggesting its role as an early-responsive gene to iron overload. Notably, *ZNF185* remained upregulated (log2 Foldchange = 1.06) in the 1.5 mM/12 h group, although the increase did not reach statistical significance (*P* = 0.24). This observation highlighted the time-dependent activation of *ZNF185* under iron overload conditions. Furthermore, *ZNF185* was significantly upregulated even at the highest iron concentration (5.0 mM/12 h and 5.0 mM/24 h), despite not being the most differentially expressed gene in these groups. Pathway enrichment analysis of the low-concentration groups (1.0 mM/24 h and 1.5 mM/24 h) revealed involvement in biological processes such as regulation of the cell cycle, organization of the microtubule cytoskeleton, and developmental processes (Fig. S1A, B). While the pathway enrichment analysis in the 5.0 mM/24 h group was associated with cell adhesion, positive regulation of programmed cell death, and negative regulation of cell population proliferation (Fig. S1C), which collectively supported the potential regulatory function of ZNF185 in the early stages of iron overload-induced granulosa cell apoptosis.

Moreover, subsequent validation experiments demonstrated dose-dependent upregulation of ZNF185 at both transcriptional and translational levels. Quantitative PCR analysis confirmed a positive correlation between FAC concentration and *ZNF185* mRNA expression (Fig. 3C). Western blot analysis similarly showed significant protein-level induction of ZNF185 following FAC treatment (Fig. 3D, E). To evaluate clinical relevance, we collected follicular fluid from both the affected ovary and the contralateral healthy ovary of unilateral ovarian endometrioma patients. Notably, *ZNF185* mRNA levels were significantly elevated in follicular fluid from endometrioma-affected ovaries compared to contralateral unaffected ovaries (Fig. 3F), corroborating our in vitro findings and suggesting pathological relevance in human ovarian disorders.

As a cytoskeleton-associated protein, ZNF185 primarily functions through interaction with F-actin. Immunofluorescence microscopy revealed profound cytoskeletal disorganization following 24-h FAC exposure, characterized by loss of the characteristic filamentous F-actin network (Fig. 3G). Furthermore, we established a stable cell line (KGN) overexpressing *ZNF185* (Fig. 3H, I). Immunofluorescence microscopy revealed that the overexpression group exhibited disorganized F-actin structures, characterized by the loss of the well-aligned normal fibrous network (Fig. 3J). These data collectively demonstrated that iron overload robustly induced *ZNF185* expression in granulosa cells, leading to significant disruption of the F-actin cytoskeleton, which may thereby initiate apoptotic signaling cascades.

### Iron overload-induced granulosa cell apoptosis through cytoskeletal remodeling and Drp1-mediated mitochondrial fission

**Fig. 4:**
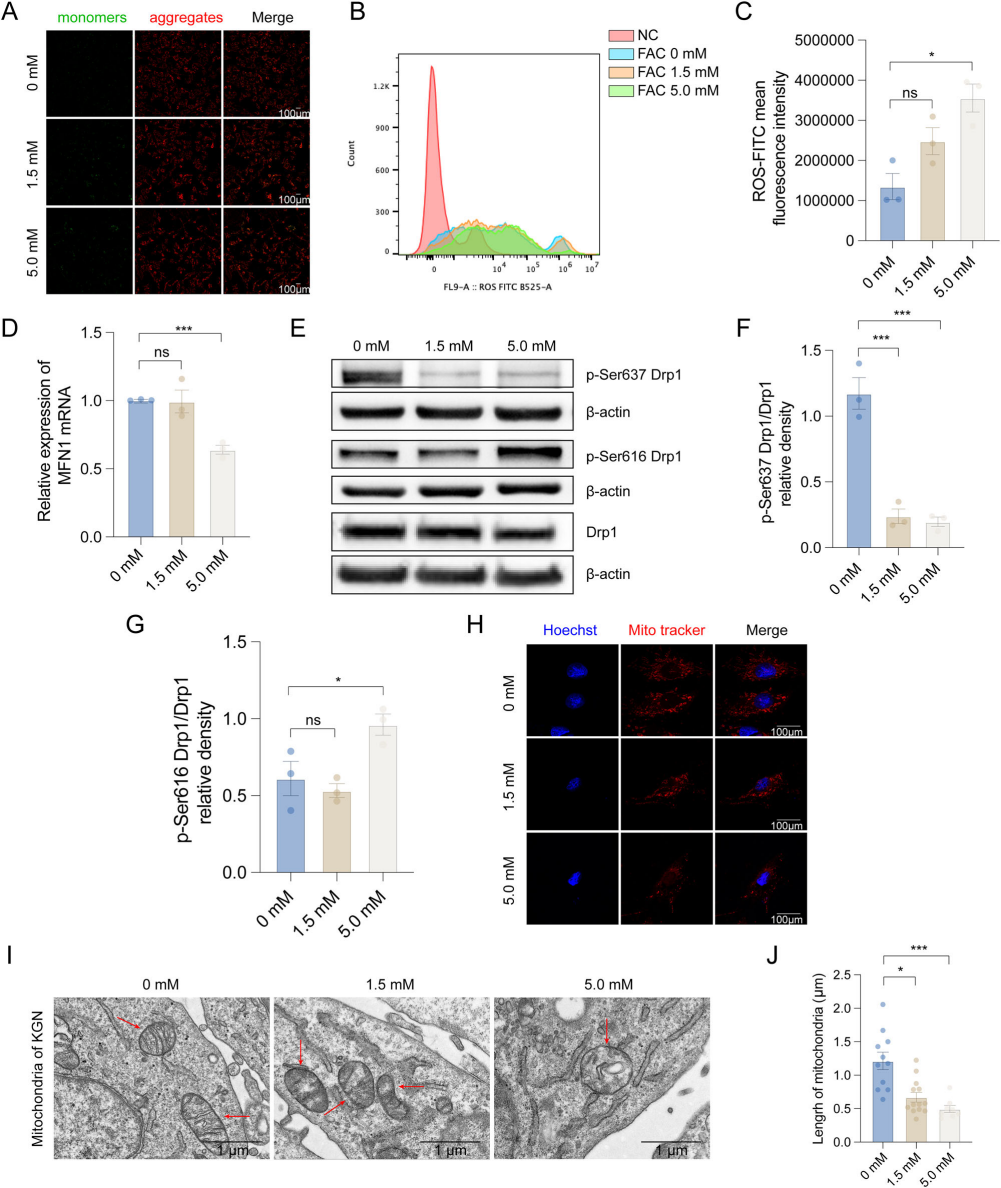
Mitochondrial dysfunction caused by iron overload in KGN cells. **A** MMP was measured by tetraethyl benzimidazole-carbocyanine iodide (JC-1) staining. JC-1 monomers (green) and JC-1 aggregates (red) were exhibited. **B**, **C** ROS levels in iron-overloaded KGN cells were measured by flow cytometry. **D** Real-time PCR validation of mitochondrial fusion-related gene *MFN1* in an iron overload circumstance (*n* = 3). **E**–**G** Comparison of p-Ser637 Drp1/Drp1, p-Ser616 Drp1/Drp1 in KGN with FAC-treated and the control group (*n* = 3). **H** Effects of iron overload on mitochondrial morphology in KGN cells. **I**, **J** The mitochondria morphology and length in FAC-stimulated KGN cells were observed. Statistical analysis was performed using one-way analysis of variance followed by the Bonferroni test. Data was shown as mean ± S.E.M. *P*-values were reported using GraphPad: not significant (ns), *P* > 0.05; *P < 0.05; **P < 0.01; ***P < 0.001. Bar (laser-scanning confocal microscopy) = 100 μm. Bar (Transmission electron microscopy) = 1 μm.

The F-actin cytoskeleton forms an intricate filamentous network that orchestrates subcellular architecture, facilitating organelle positioning, intracellular transport, and inter-organelle communication. This structural framework is particularly critical for maintaining mitochondrial homeostasis and function. Mitochondrial membrane potential (ΔΨm), the electrochemical gradient across the inner mitochondrial membrane generated by the electron transport chain, serves as a key indicator of mitochondrial health. Depolarization of ΔΨm represents an early and irreversible commitment to apoptotic cascades, preceding cytochrome C release and caspase activation. To quantitatively assess ΔΨm dynamics in our iron overload model, we employed the JC-1 fluorescent probe. KGN cells were collected after 1.5 mM/12 h FAC treatment, followed by the measurement of the relative ratio of red and green fluorescence. The results showed that the JC-1 monomer rate (green fluorescence) increased after FAC treatment (Fig. 4A). Next, we measured the ROS levels in KGN cells after FAC treatment. The average fluorescence intensity of ROS-FITC increased significantly with increasing FAC concentration (Fig. 4B, C).

The dynamic equilibrium between actin polymerization (F-actin formation) and depolymerization (G-actin) serves as a critical regulator of mitochondrial morphology and function. Mechanistically, polymerized F-actin facilitates the translocation of dynamin-related protein 1 (Drp1) from the cytosol to mitochondrial scission sites, where it oligomerizes to form constrictive rings that drive mitochondrial fission. Therefore, we first examined the expression levels of mitochondrial fusion-related genes in KGN cells under iron overload. The mRNA expression level of Mitofusion1 (*MFN1*) in the iron overload group significantly decreased (Fig. 4D). In addition, the phosphorylation level of serine 616 residue of the mitochondrial fission-related protein Drp1 increased, while the phosphorylation level of serine 637 residue decreased, indicating the activation of Drp1 protein and the stimulation of mitochondrial fission (Fig. 4E–G).

Furthermore, we labeled the morphology of mitochondria in FAC-stimulated KGN cells and performed imaging using laser-scanning confocal microscopy. The mitochondria of the control group exhibited various shapes, such as spherical, rod-like, or filamentous, while mitochondria of the FAC-stimulated group were in spotty shapes, along with the reduced intensity of red fluorescence (Fig. 4H). Transmission electron microscopy showed that mitochondria in the FAC-stimulated group exhibited a significantly swollen shape, disappearance of cristae structures, and shortened mitochondrial length (Fig. 4I, J). These findings collectively demonstrate that iron overload perturbs mitochondrial dynamics by suppressing MFN1-mediated fusion while promoting Drp1-dependent fission, ultimately leading to pathological mitochondrial fragmentation.

### Iron overload-induced endoplasmic reticulum (ER) stress in granulosa cells

**Fig. 5:**
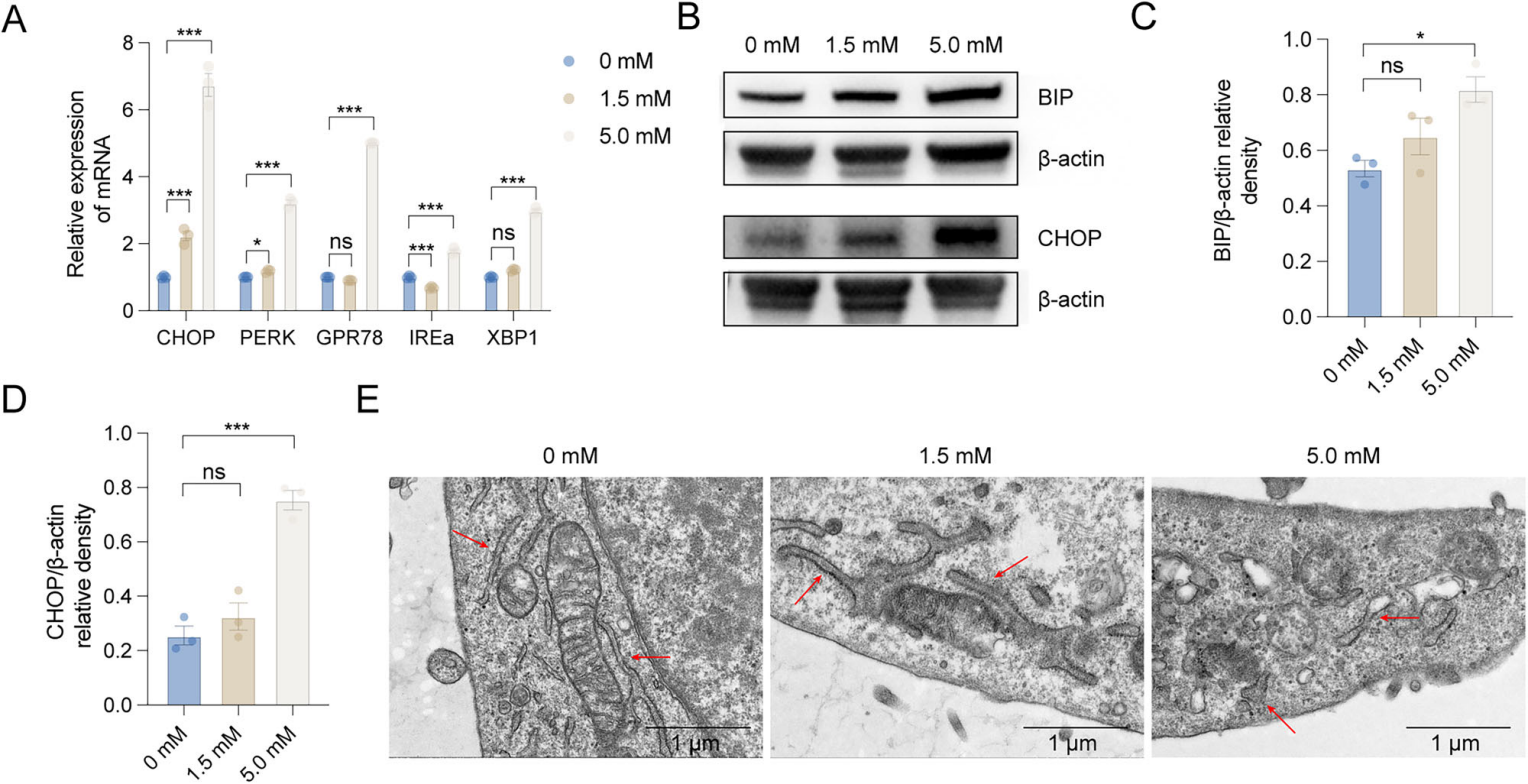
Endoplasmic reticulum stress caused by iron overload in KGN cells. **A** Real-time PCR validation of representative ER stress-related genes (*n* = 3). **B**–**D** Comparison of *BIP* and *CHOP* in KGN with FAC treatment and the control group (*n* = 3). **E** The endoplasmic reticulum morphology in FAC-stimulated KGN cells was observed. Statistical analysis was performed using one-way analysis of variance followed by the Bonferroni test. Data was shown as mean ± S.E.M. *P*-values were reported using GraphPad: not significant (ns), *P* > 0.05; *P < 0.05; **P < 0.01; ***P < 0.001. Bar = 1 μm.

Emerging evidence indicates that F-actin polymerization modulates calcium ion (Ca^2+^) release from the endoplasmic reticulum (ER). Specifically, the actin cytoskeleton facilitates Ca^2+^ transfer to mitochondria at ER-mitochondria contact sites (MERCs), which can induce mitochondrial dysfunction, ROS overproduction, and aggravate ER stress. To investigate this pathway in our model, we quantified ER stress-related gene expression in FAC-treated KGN cells using qRT-PCR. Quantitative analysis revealed significant upregulation of key ER stress markers at the transcriptional level, including *CHOP* (*DDIT3*), *PERK* (*EIF2AK3*), *GRP78* (*HSPA5*), *IRE1α* (*ERN1*), and *XBP1* in FAC-treated KGN cells (Fig. 5A). Consistent with mRNA findings, immunoblotting demonstrated markedly elevated protein expression of the ER chaperone BIP (GRP78) and pro-apoptotic transcription factor CHOP (Fig. 5B–D). Ultrastructural examination by transmission electron microscopy revealed characteristic ER pathology, exhibiting dilated cisternae and prominent swelling in iron-overloaded cells (Fig. 5E). These morphological alterations, indicative of severe ER dysfunction, may activate apoptotic pathways through the PERK-CHOP signaling axis.

### *ZNF185* knockdown attenuated iron overload-induced granulosa cell apoptosis through cytoskeletal stabilization

**Fig. 6:**
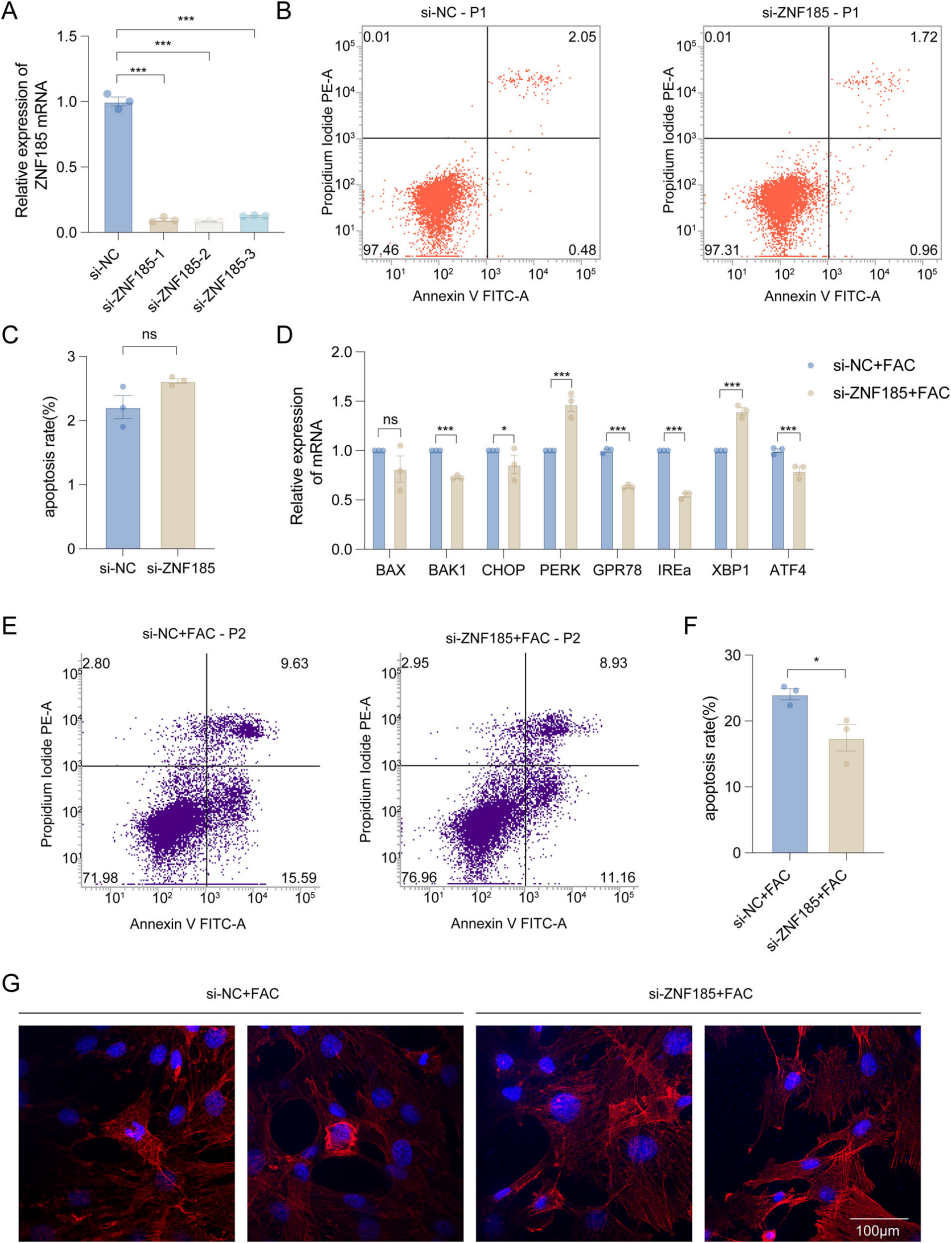
Rescue effect of *ZNF185* knockdown on KGN under iron overload. **A**–**C** Three si-*ZNF185* RNA was transfected into KGN cells. Then the *ZNF185* expression and apoptosis rates were examined through qRT-PCR and flow cytometry, respectively (*n* = 3). **D** Real-time PCR validation of representative apoptosis and ER stress-related gene (*n* = 3) in KGN after FAC treatment. **E**, **F** The apoptosis rate of KGN cells under various FAC concentrations for 24 h after transfection of si-*ZNF185* RNA (*n* = 3). **G** Morphology of cytoskeleton F-actin with iron overload was exhibited after transfection of si-*ZNF185* RNA. Statistical analysis was performed using Student’s *t*-test (two groups) and one-way analysis of variance followed by the Bonferroni test (more than two groups). Data was shown as mean ± S.E.M. *P*-values were reported using GraphPad: not significant (ns), *P* > 0.05; *P < 0.05; **P < 0.01; ***P < 0.001. Bar = 100 μm.

To mechanistically characterize ZNF185's role in FAC-induced apoptosis, we designed three specific siRNAs targeting *ZNF185*, all of which achieved efficient knockdown (Fig. 6A). Under basal conditions, *ZNF185* depletion did not significantly affect KGN cell viability (Fig. 6B, C), suggesting its apoptotic role is stress-dependent. Next, we investigated whether *ZNF185* knockdown could alleviate the damage caused by FAC in KGN cells. Using RT-qPCR analysis, we demonstrated that *ZNF185* knockdown significantly decreased the mRNA expression levels of pro-apoptotic genes *BAX* and *BAK1* (Fig. 6D). Additionally, knockdown of *ZNF185* reduced the expression of ER stress markers *CHOP*, *GRP78*, *IRE1α,* and *ATF4*, and increased the expression of *PERK* and *XBP1*, indicating that endoplasmic reticulum stress has been alleviated to some extent (Fig. 6D).

**Fig. 7:**
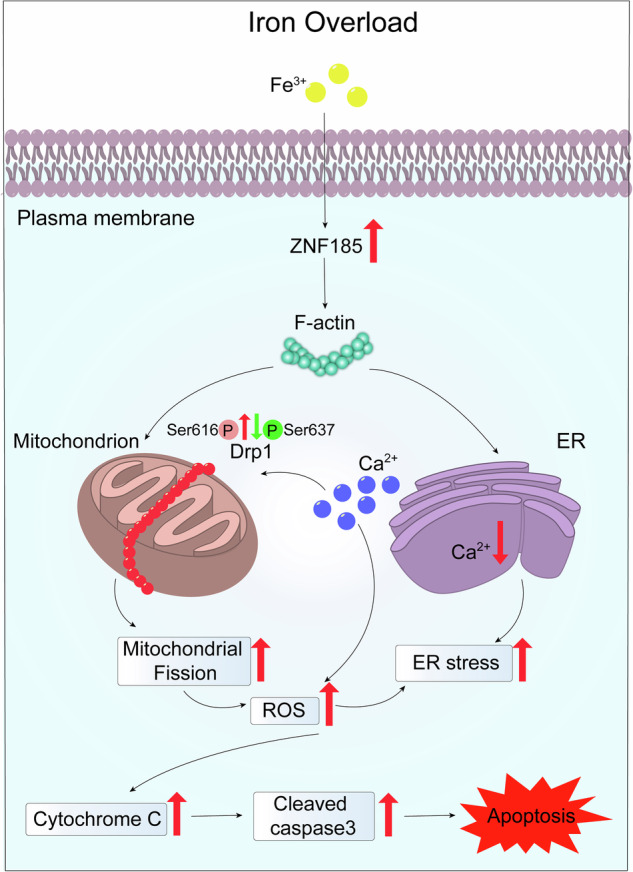
Graphical abstract summarizing the effects and mechanisms of *ZNF*185 induction of apoptosis in KGN cells under iron overload conditions. *ZNF185* led to the recruitment of Drp1 enzyme, excessive mitochondrial division, and an increase in ROS levels, ultimately resulting in apoptosis through the mitochondrial pathway. Meanwhile, *ZNF185* induced apoptosis through endoplasmic reticulum stress.

In this study, we hypothesized that downregulation of *ZNF185* could ameliorate iron overload-induced apoptosis by restoring the abnormal dynamics of F-actin cytoskeletal polymerization. Therefore, we treated *ZNF185* knockdown KGN cells with FAC for 24 h and collected the cells for flow cytometry analysis. The results revealed that *ZNF185* knockdown partially rescued FAC-induced apoptosis in KGN cells (Fig. 6E, F). Furthermore, immunofluorescence analysis of F-actin morphology in *ZNF185*-knockdown cells under iron overload conditions demonstrated that the disrupted cytoskeletal network was restored to a normal fibrous architecture compared to the control group (Fig. 6G). Collectively, these findings suggested that downregulation of *ZNF185* alleviated cytoskeletal disruption, suppressed excessive mitochondrial fission and ER stress triggered by iron overload, and mitigated apoptosis in KGN cells (Fig. 7).

## Discussion

In this study, we explored the potential mechanisms by which iron overload compromised oocyte development in patients with ovarian endometrioma. Firstly, we confirmed that patients with ovarian endometrioma exhibited decreased AMH levels, reduced follicle count, fewer retrieved oocytes, and elevated FSH, indicating diminished ovarian reserve. Next, we demonstrated that iron overload-induced excessive mitochondrial fission, cytochrome C release, and subsequent Caspase-3 activation, along with endoplasmic reticulum (ER) stress, ultimately leading to granulosa cell apoptosis. Notably, our findings identified ZNF185 as a novel iron-responsive regulator that mediated iron overload-induced cellular dysfunction. Mechanistically, iron overload significantly upregulated *ZNF185* expression, which in turn disrupted F-actin cytoskeletal architecture and subsequently compromised both mitochondrial and endoplasmic reticulum homeostasis. Knockdown of *ZNF185* partially restored cytoskeletal morphology and reduced granulosa cell apoptosis. Our study elucidated a novel mechanism underlying the decline in ovarian reserve in patients with ovarian endometrioma and provided a potential molecular therapeutic target for fertility preservation in these patients.

*ZNF185*, located on chromosome Xq28, belongs to a zinc finger protein containing a C-terminal LIM domain. It co-localizes with F-actin and plays critical roles in cellular development, differentiation, and carcinogenesis [18]. Studies suggest that *ZNF185* is downregulated in multiple epithelial cancers (e.g., prostate, lung, and pancreatic cancers) [19], likely due to its transcriptional activation by wild-type p53 upon DNA damage [20]. This activation suppresses tumor progression via cytoskeletal remodeling, implicating *ZNF185* as a tumor suppressor [21]. To the best of our knowledge, this is the first study investigating the impact of *ZNF185* in the reproductive system. Iron overload upregulated *ZNF185* expression in granulosa cells and triggered F-actin remodeling, which was followed by Drp1 recruitment, excessive mitochondrial fission, ROS accumulation, ER stress, and ultimately granulosa cell apoptosis. A study has shown that siRNA-mediated *ZNF185* knockdown in Ker-CT cells treated with etoposide showed no significant changes in cell cycle distribution or apoptosis [20]. Previous studies have reported that *ZNF185* depletion induced actin filament disassembly and enhanced stress fiber formation in endothelial cells [17]. In contrast, our findings revealed that while *ZNF185* knockdown does not significantly affect basal apoptosis rates in granulosa cells (*P* > 0.05), suggesting that *ZNF185* may play cell-type-specific roles in the regulation of apoptosis. This discrepancy highlights the potential for *ZNF185* to exert distinct functional impacts across different cellular contexts, thereby contributing to the complexity of its biological role in cellular homeostasis and apoptosis regulation. Furthermore, our study demonstrated that *ZNF185* conferred partial protection against iron overload-induced cytoskeletal disruption and subsequent apoptosis. To further elucidate the pathophysiological role of *ZNF185* in ovarian iron homeostasis in vivo, the generation of granulosa cell-specific *ZNF185* knockout mouse models will be critical for investigating its impact on follicular development and ovarian reserve under iron overload conditions.

We observed that iron overload upregulated *ZNF185* expression, which co-localized with F-actin and induced F-actin dynamic disorganization, leading to excessive recruitment of activated Drp1 to mitochondria. This promoted mitochondrial hyperfission, respiratory dysfunction, increased cytochrome C release, elevated ROS production, and ultimately apoptosis. It was reported that the C-terminal LIM domain of ZNF185, containing Lin-11, Isl-1, and Mec-3 (LIM) motifs, primarily interacts with focal adhesions [22]. Although focal adhesions in granulosa cells remain understudied, emerging evidence suggests their crucial roles in granulosa cell-oocyte communication [23], ovulation [24], and apoptosis [25]. Therefore, we propose that investigating whether iron overload affects granulosa cell function and apoptosis through ZNF185-mediated focal adhesion modulation represents an intriguing research direction. Furthermore, identifying proteins that interact with other domains of ZNF185 will help elucidate the mechanisms underlying its diverse subcellular localizations and functions.

The therapeutic efficacy of targeting the cytoskeleton in cancer has been fully demonstrated [18]. Currently, the only cytotoxic agents specifically targeting microtubules used in chemotherapy are microtubule stabilizers and microtubule polymerization inhibitors [26, 27]. However, there is a lack of effective, specific-targeting drugs for other components of the cytoskeleton, such as actin, intermediate filaments, and other signaling molecules involved in cytoskeletal regulation. Although potential actin-specific chemotherapy drugs like cytochalasin and MKT-077 might have application prospects [28], their non-specific targeting may lead to significant cytotoxicity due to the crucial role of actin in normal cellular functions [29, 30]. Therefore, it is imperative to develop highly selective actin-targeting drugs. Our study elucidates a novel mechanism underlying the decline in ovarian reserve in patients with ovarian endometrioma and provides a potential molecular therapeutic target for fertility preservation in these patients.

However, this study still has certain limitations, as the mechanistic investigations were confined to the cellular level. Therefore, a mouse model overexpressing the *ZNF185* gene can be established to investigate whether it leads to increased apoptosis of granulosa cells, decreased ovarian reserve function, and abnormal oocyte development in mice. However, the underlying mechanism by which iron overload induces the upregulation of *ZNF185* expression remains unexplored in this study and needs further experimental validation.

## Conclusion

This study reveals that iron overload contributes to the decline in ovarian reserve in patients with ovarian endometrioma by inducing mitochondrial dysfunction, ER stress, and granulosa cell viability loss. We identify ZNF185 as a novel iron-responsive gene that orchestrates these effects through F-actin cytoskeleton disruption, leading to impaired organelle homeostasis. Critically, ZNF185 knockdown attenuates iron-induced cellular damage and apoptosis, suggesting its therapeutic potential for preserving fertility in women with ovarian endometrioma.

## Materials and methods

### Study population

This study conducted a retrospective analysis of clinical data from patients who underwent in vitro fertilization/intracytoplasmic sperm injection-embryo transfer (IVF/ICSI-ET) treatment at the Reproductive Center of Sun Yat-sen Memorial Hospital of Sun Yat-sen University between January 2016 and November 2021. The inclusion criteria for the ovarian endometrioma group were as follows: age <35 years; diagnosis of unilateral or bilateral ovarian endometrioma by transvaginal ultrasound; and no prior surgical treatment. The exclusion criteria were: (1) concomitant other ovarian pathologies, including ovarian tumors, ovarian agenesis, ovarian cysts, or previous surgical removal of endometriomas; (2) severe tubal effusion; (3) polycystic ovary syndrome. Concurrently, patients of comparable ages with infertility due to male factors were selected as the control group. Ultimately, 712 control patients and 187 patients with ovarian endometrioma were included.

### Isolation of granulosa cells from follicular fluid

To evaluate the expression of the *ZNF185* gene in granulosa cells of ovarian endometrioma patients, we collected follicular fluid from four patients who underwent in vitro fertilization/intracytoplasmic sperm injection-embryo transfer (IVF/ICSI-ET) treatment at the Reproductive Center of Sun Yat-sen Memorial Hospital of Sun Yat-sen University. Each patient had an ovary with an endometrioma and the contralateral healthy ovary. The follicular fluid from the first largest follicle of each ovary was collected separately. Then, the sample was centrifuged at 350 × *g* for 10 min, followed by density gradient centrifugation with Lymphocyte Separation Medium (LTS1077, TBD, Tianjing, China) at 350 × *g* for 15 min. The middle layer containing cellular components was collected, washed with PBS, and centrifuged again at 300 × *g* for 5 min to obtain the pellet. The isolated granulosa cells were then lysed for subsequent qPCR experiments.

### Cell culture and iron overload model establishment in vitro

KGN cell lines were purchased from Procell System Company (CL-0603, Wuhan, China). Short tandem repeat (STR) testing was performed to confirm cell identity. KGN cell lines were tested negative for mycoplasma contamination, cultured in Dulbecco’s modified Eagle’s medium Ham’s F12 (C11995500BT, GIBCO, California, USA) supplemented with 10% fetal bovine serum (FSP500, ExCell Bio, Shanghai, China) and incubated at 37 °C and 5% CO_2_. According to our previous study [16], 1.5 mM and 5.0 Ferric ammonium citrate (FAC) (D8517, Sigma–Aldrich, USA) were used for building the iron overload model. Then the KGN cells were collected for the following apoptosis analysis, cell viability, MMP, and ROS assay.

### Construction of a KGN cell line with stable overexpression of ZNF185

Lentiviral products were obtained by co-transfection of ZNF185 plasmids with two viral packaging plasmids into HEK293T using the Lipo3000 (L3000008, Thermo Fisher, USA) method. After 72 h of transfection, the virus supernatant was harvested and passed through a 0.45 μm filter. Then, the KGN cell line was seeded in six-well plates and the next day transduced with a mixture of DMEM/F12 and virus supernatant at a ratio of 1:1. After 12–16 h of incubation, the cells were selected in the presence of puromycin for 3 days.

### Real-time PCR (qPCR)

EZ-press RNA Purification Kit (B0004D, EZB, USA) was used to extract the total RNA of KGN cells according to the manufacturer’s protocol. The RNA was then transformed into cDNA using the HiScript III RT SuperMix for qPCR (R323-01, Vazyme, Nanjing, China). The PCR was conducted using the LightCycler^®^ 96 system with ChamQ Universal SYBR qPCR Master Mix (Q711-02, Vazyme, Nanjing, China). The relative RNA expression was quantified by normalizing the cycle threshold (Ct) values of target genes, comparing it to *GAPDH,* and then calculating by the 2-△△CT method; △Ct = Avg. Ct sample- Avg. Ct GAPDH; △△Ct = Avg.△Ct Endometriosis -Avg.△Ct controls. The primer sequence of the qPCR assay used in this study is provided in Table S2.

### Western blot analysis

Protein extraction lysis was composed of RIPA lysis buffer (FD009, Fdbio science, Pennsylvania, USA) supplemented with 1% Protease Inhibitor Cocktail (CW2200S, CWBIO, Jiangsu, China) and 1% Phosphatase Inhibitor Cocktail (CW2383S, CWBIO). KGN cells were lysed with ice-cold Protein extraction lysis for 30 min. Protein concentration was then measured using a BCA protein assay kit (CW0014S, CWBIO). 25 μg protein of each sample was loaded and separated in 10% SDS-PAGE (F15420L, ACE, Shanghai, China) according to the manufacturer’s instructions. Protein samples were then blotted onto PVDF membranes (IPVH00010, Merck, Germany), which were then blocked with 5% defatted milk and incubated overnight with primary antibodies at 4 °C. β-actin was used in each run as an internal control. Primary antibodies used are as follows: β-actin (1:1000, GB11001, Servicebio, Wuhan, China), Caspase-3 (1:1000, A19654, ABclonal, Wuhan, China), CYC (1:1000, A4912, ABclonal), p-Ser616 Drp1 (1:1000, AP1353, ABclonal), p-Ser637 Drp1 (1:500, AF5791, Beyotime, Shanghai, China), Drp1 (1:4000, A21968, ABclonal), CHOP (1:1000, 2895T, CST, Massachusetts, USA), BIP (1:6000, A23453, ABclonal), ZNF185 (1:1000, NBP1-86452, Colorado, Novus Biologicals, USA). The protein expression was quantified with ImageJ software. The image of full-length western blots is in Supplementary Material 2.

### Fluorescent staining of the cytoskeleton F-actin

KGN cells after treatments were washed with PBS twice and fixed for 15 min at room temperature with 4% paraformaldehyde. For cytoskeleton F-actin staining only, the above fixed cells were permeabilized for 5 min at room temperature with permeabilization buffer (0.1% Triton X-100 in PBS) three times. Then, cells were incubated at room temperature in the dark for 30 min with Rhodamine Phalloidin (RM02835, Abclonal, 1:50 in PBS) or SF488 Phalloidin (CA1640, Solarbio, Beijing, China). After incubation, cells were washed three times with PBS and mounted with DAPI (C0065-10, Solarbio) for 10 min. All fluorescent images were captured using a confocal microscope (Olympus FV3000, Japan).

### Cell viability assay

KGN cells were seeded in 96-well plate (TCP010096, Jet, Guangzhou, China) at a density of 5000 cells. After treating with/without 1.5 mM/24 h and 5.0 mM/24 h FAC, cell viability was assessed by cell counting kit-8 assays (CCK8-K1018-5, APExBIO, State of Texas, USA) by the manufacturer’s protocols. The signals were measured by the Bio-Tek SynergyH1 system.

### Apoptosis analysis

At 24 h post FAC treatment, KGN cells were collected and stained using an Annexin V-FITC/PI Apoptosis Detection Kit (A211, Vazyme) according to the manufacturer’s procedures.

### RNA sequencing

A multi-concentration and multi-timepoint experimental design was employed to systematically characterize iron overload effects. Total RNA was isolated from five treatment groups of KGN cells: 1.0 mM/24 h, 1.5 mM/12 h, 1.5 mM/24 h, 5.0 mM/12 h, and 5.0 mM/24 h, as per the manufacturer’s protocols (B0004D, EZB, USA). The quality of isolated RNA was examined by NanoDrop ND-1000 (NanoDrop, DE, USA) and Agilent 2100 Bioanalyzer (Agilent, Santa Clara, CA). The qualified samples were then subjected to paired-end library preparation according to the Illumina protocol (Illumina, San Diego, CA). The libraries were sequenced to produce 150 paired-end reads on an Illumina NovaSeq sequencing platform. These data were deposited into the National Center for Biotechnology Information (NCBI) Sequence Read Archive (SRA) under accession number PRJNA1287648.

### Detection of ROS

After FAC treatment for 12 h, 10^6^ KGN cells were collected and incubated with DCFH-DA (2′,7′-dichlorofluorescin diacetate) (S0033S, Boytime) for 20 min at 37 °C in the dark. Then, cells were washed with Opti-DMEM/F12 three times, followed by flow cytometry quantification. All images were visualized using by software FlowJo.

### Measurement of mitochondrial membrane potential (MMP)

10^6^ KGN cells were collected after FAC treatment for 12 h. MMP was measured by tetraethyl benzimidazole-carbocyanine iodide (JC-1) (C2006, Beyotime) staining according to the manufacturer’s protocol. All fluorescent images were captured using a confocal microscope (Olympus FV3000).

### Fluorescent staining of mitochondria

1 × 10^6^ KGN cells were collected after FAC treatment for 12 h and were incubated with Mito-Tracker Red (C1035, Beyotime) and Hoechst 33342 (C0031, Beyotime) according to the manufacturer’s procedure. All fluorescent images were captured using a confocal microscope (Olympus FV3000).

### Transmission electron microscopy of KGN

KGN cells were seeded in 10 cm cell culture dishes at a density of 5 × 10^5^ cells. After treating with/without 1.5 mM/24 h and 5.0 mM/24 h FAC, KGN cells were collected by cell scraper and collected in Eppendorf tubes. The cell pellets were suspended and fixed with 2.5% glutaraldehyde (P1126, Servicebio) at 4 °C overnight. The fixed cells were washed in 0.1 M phosphate buffer (pH 7.4) 3 times for 3 min each. A 2% low-melting-point agarose solution was prepared by heating and dissolving it in advance. After being cooled to about 40 °C, the agarose solution was added to wrap the cell pellets. Subsequently, the cells were dehydrated with a graded series of ethanol and gradually penetrated with resin. Then, the cells were embedded in pure LR white resin (Ladd Research Industries, Burlington, VT). The ultrathin sections were cut to 70–80 nm thickness on an ultramicrotome (Leica, UC7) and mounted on 150 mesh nickel grids. The sections were subjected to double staining with uranyl acetate and lead citrate before being observed under a transmission electron microscope (Hitachi HT7800).

### RNA interference

siRNA targeting the *ZNF185* gene (siRNA1: UCUAGGGUAAUCUUUGGAC, siRNA: UUCACUAGCAUUCACGUAC, siRNA3: GGAGGGAUCUGUACUUACU) was synthesized from RIBOBIO (Guangzhou, China). KGN cells were seeded in a 6-well cell culture plate and incubated overnight before transfection. The incubation medium was changed to DMEM/F12 without serum before transfection. Subsequently, siRNA (25 pmol) and Lipofectamine RNAimax (1.5 μL, 13778150, Thermo Fisher) were used according to the manufacturer’s protocol. The knockdown of *ZNF185* expression level was confirmed at 24 h with RT-PCR.

### Statistics analysis

Samples (cells) were randomly assigned to experimental groups utilizing a random sampling methodology, ensuring a minimum of three replicates per group to enhance the robustness of the findings. To further minimize potential bias, the allocation process was conducted blindly, and the analysis results were independently scrutinized by multiple investigators. The experimental data was analyzed with IBM SPSS Statistics 20. Student’s t-test was performed for comparisons between the two groups. For comparisons of more than two groups, statistical analysis was performed using One-way ANOVA and the Bonferroni test, respectively. Data were shown as the mean ± S.E.M. *P*-values were reported using GraphPad: not significant (ns), *P* > 0.05; *P < 0.05; **P < 0.01; ***P < 0.001.

## Supplementary information

Supplementary Material 1

Supplementary Material 2

Supplementary Table1

## Data Availability

All remaining data that support the findings of this study are available from the corresponding author (ZQX) upon request.
