# Supplementary material for: Iron-responsive ZNF185 overexpression drives mitochondrial fission and endoplasmic reticulum stress via cytoskeletal remodeling in granulosa cells
Source: Cell Death Discov. 2025 Aug 28;11:414. doi: 10.1038/s41420-025-02719-y (PMC12394633; doi:10.1038/s41420-025-02719-y)
Supplement: Supplementary file 1 — Supplementary Material 1 [file 41420_2025_2719_MOESM1_ESM.docx]

**Table S2. The sequences of qPCR primers in this study.**

| Gene name | | Forward primer (5’-3’) | Reverse primer (5’-3’) |
| --- | --- | --- | --- |
| *BAX* | TCAGGATGCGTCCACCAAGAAG | | TGTGTCCACGGCGGCAATCATC |
| *BAK1* | TTACCGCCATCAGCAGGAACAG | | GGAACTCTGAGTCATAGCGTCG |
| *ZNF185* | AGACACAGGCACCGTTTATCGC | | CTGTTTGAGCCAGGAGTGGATC |
| *MFN1* | GGTGAATGAGCGGGCTTTCCAAG | | TCCTCCACCAAGAAATGCAGGC |
| *CHOP* | GGTATGAGGACCTGCAAGAGGT | | CTTGTGACCTCTGCTGGTTCTG |
| *PERK* | GTCCCAAGGCTTTGGAATCTGTC | | CCTACCAAGACAGGAGTTCTGG |
| *GRP78* | CTGTCCAGGCTGGTGTGCTCT | | CTTGGTAGGCACCACTGTGTTC |
| *IRE1a* | CCGAACGTGATCCGCTACTTCT | | CGCAAAGTCCTTCTGCTCCACA |
| *XBP1* | CTGCCAGAGATCGAAAGAAGGC | | CTCCTGGTTCTCAACTACAAGGC |
| *ATF4* | TTCTCCAGCGACAAGGCTAAGG | | CTCCAACATCCAATCTGTCCCG |


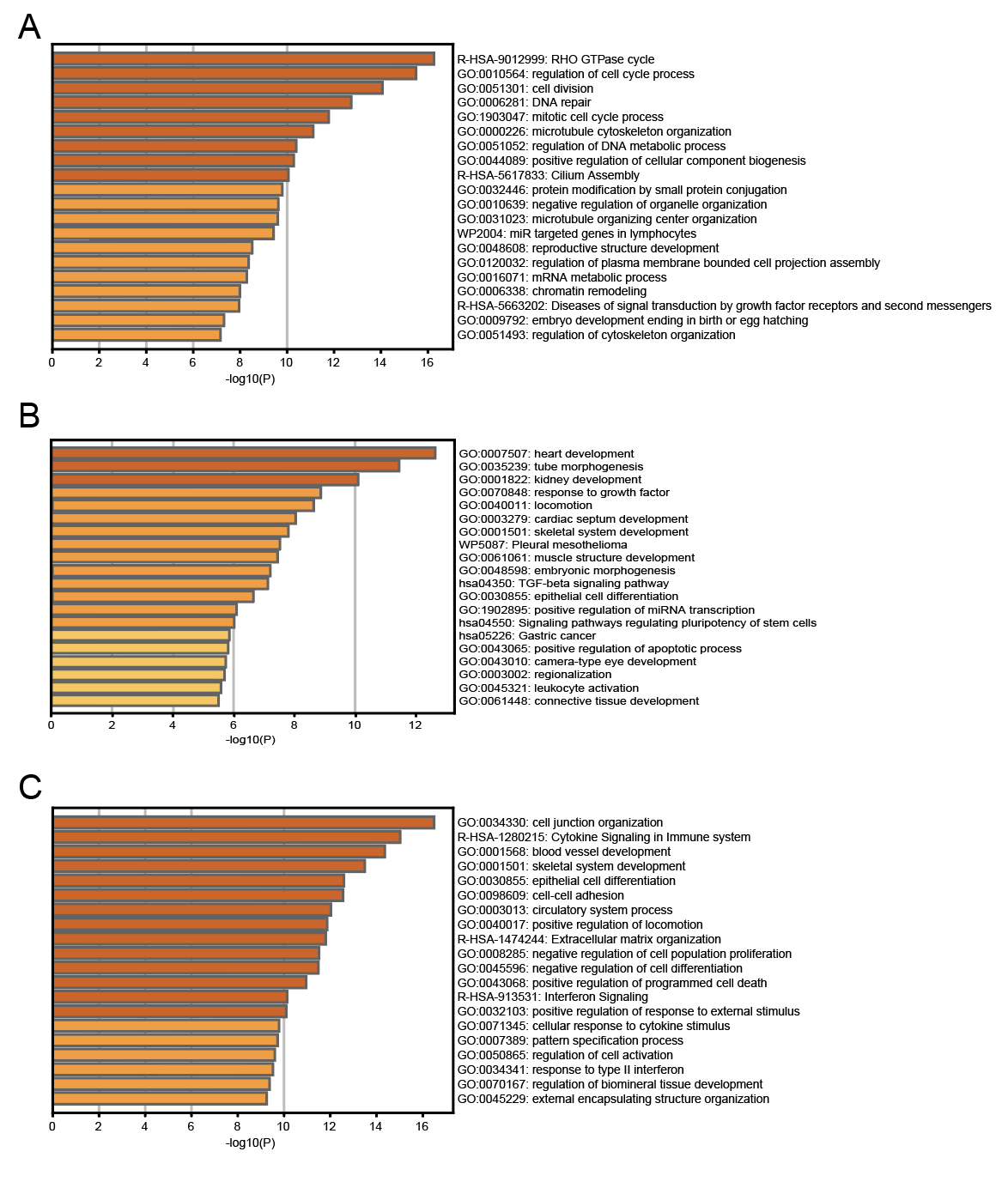


**Figure S1. GO enrichments analysis for upregulated genes in RNA sequencing of 1.0 mM/24h group (A), 1.5 mM/24h group (B) and 5.0 mM/24h group (C).**
