# Supplementary material for: Iron-responsive ZNF185 overexpression drives mitochondrial fission and endoplasmic reticulum stress via cytoskeletal remodeling in granulosa cells
Source: Cell Death Discov. 2025 Aug 28;11:414. doi: 10.1038/s41420-025-02719-y (PMC12394633; doi:10.1038/s41420-025-02719-y)
Supplement: Supplementary file 2 — Supplementary Material 2 [file 41420_2025_2719_MOESM2_ESM.docx]

**Fig. 2 Effects of iron overload on proliferation, apoptosis, and estrogen secretion in KGN cells. D** Comparison of cytochrome C and cleaved caspase-3 in KGN with FAC treated and the control group (n = 3).


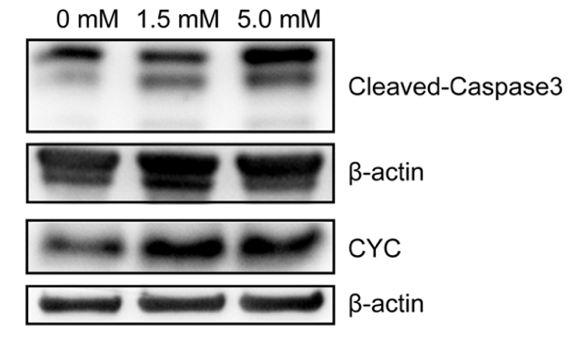


**Full length Western Blots:**

**
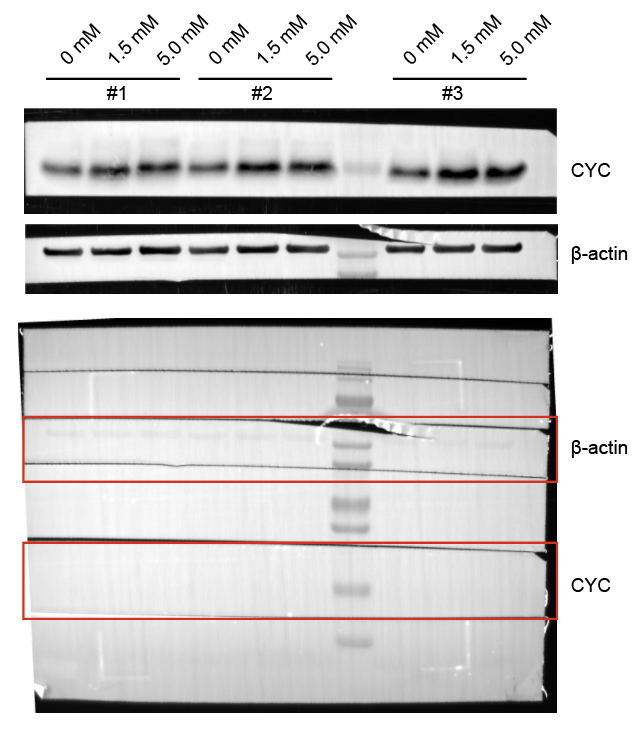
**

**
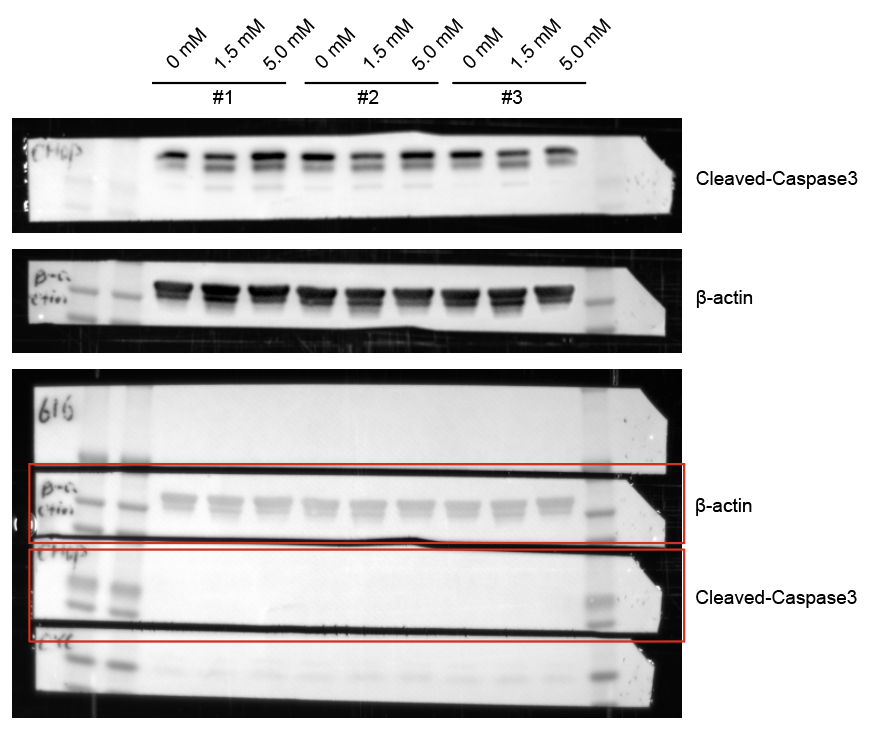
**

**Fig. 3** **The correlation between *ZNF185* and iron overload-induced apoptosis in KGN. C** Comparison of ZNF185 in KGN with FAC treated and the control group (n = 3).


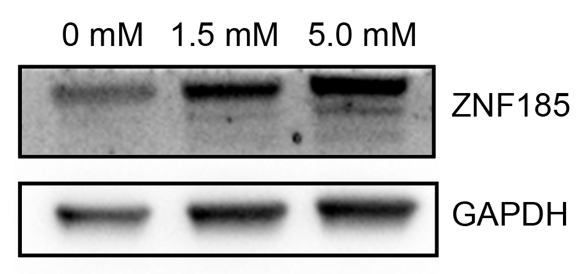


**Full length Western Blots:**

**
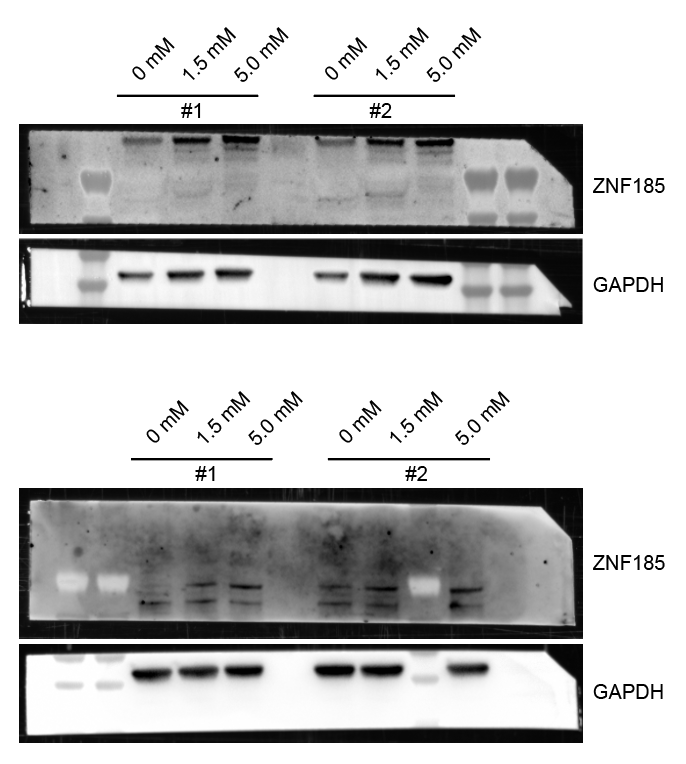
**

**Fig. 3** **The correlation between *ZNF185* and iron overload-induced apoptosis in KGN. H** Analysis of ZNF185 overexpression in stable KGN cell line by Western Blots.

**
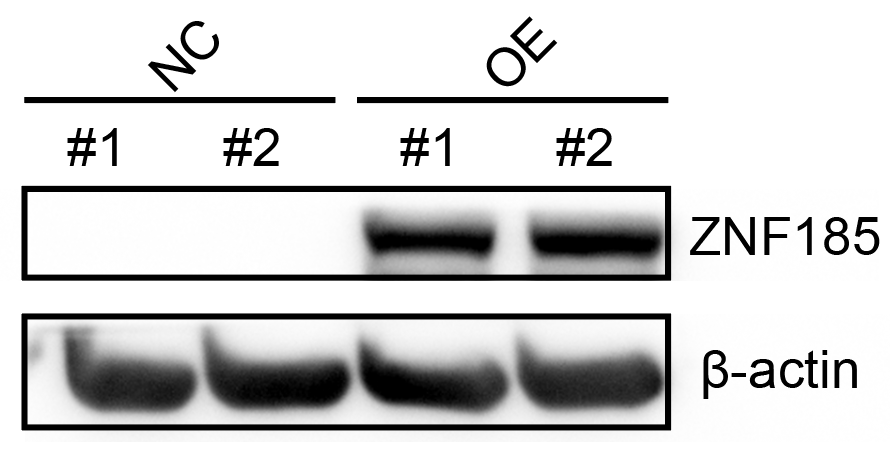
**

**Full length Western Blots:**

**
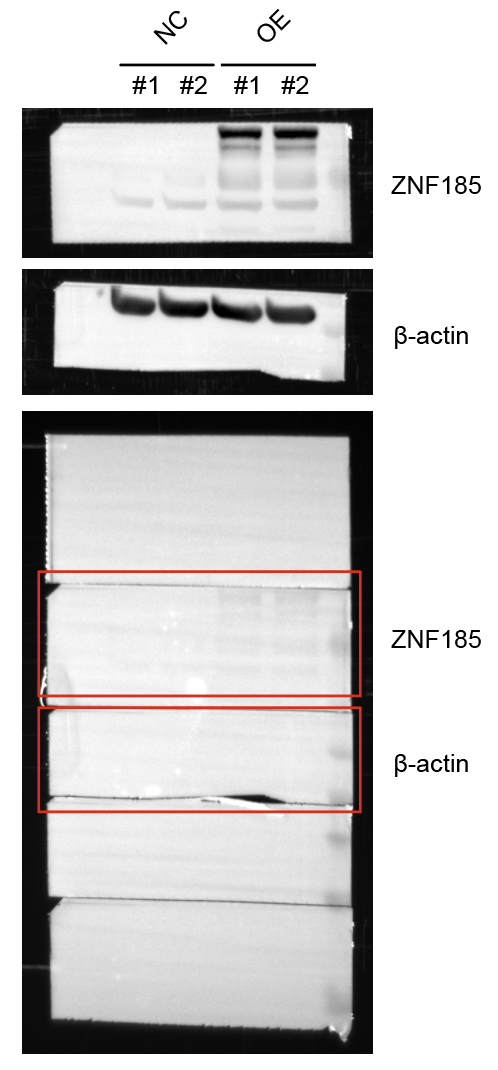
**

**Fig. 4 Mitochondrial dysfunction caused by iron overload in KGN cells. E** Comparison of p-Ser637 DRP1/ DRP1, p-Ser616 DRP1/ DRP1 in KGN with FAC treated and the control group (n = 3).

**
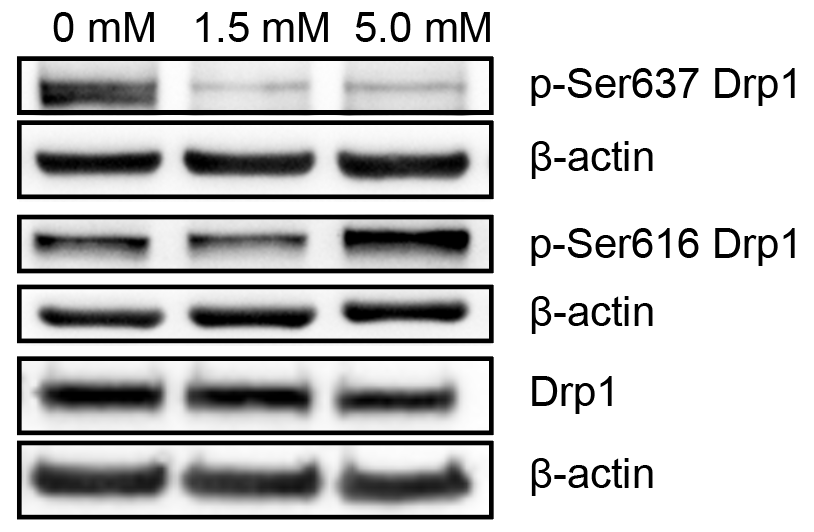
**

**Full length Western Blots:**

**
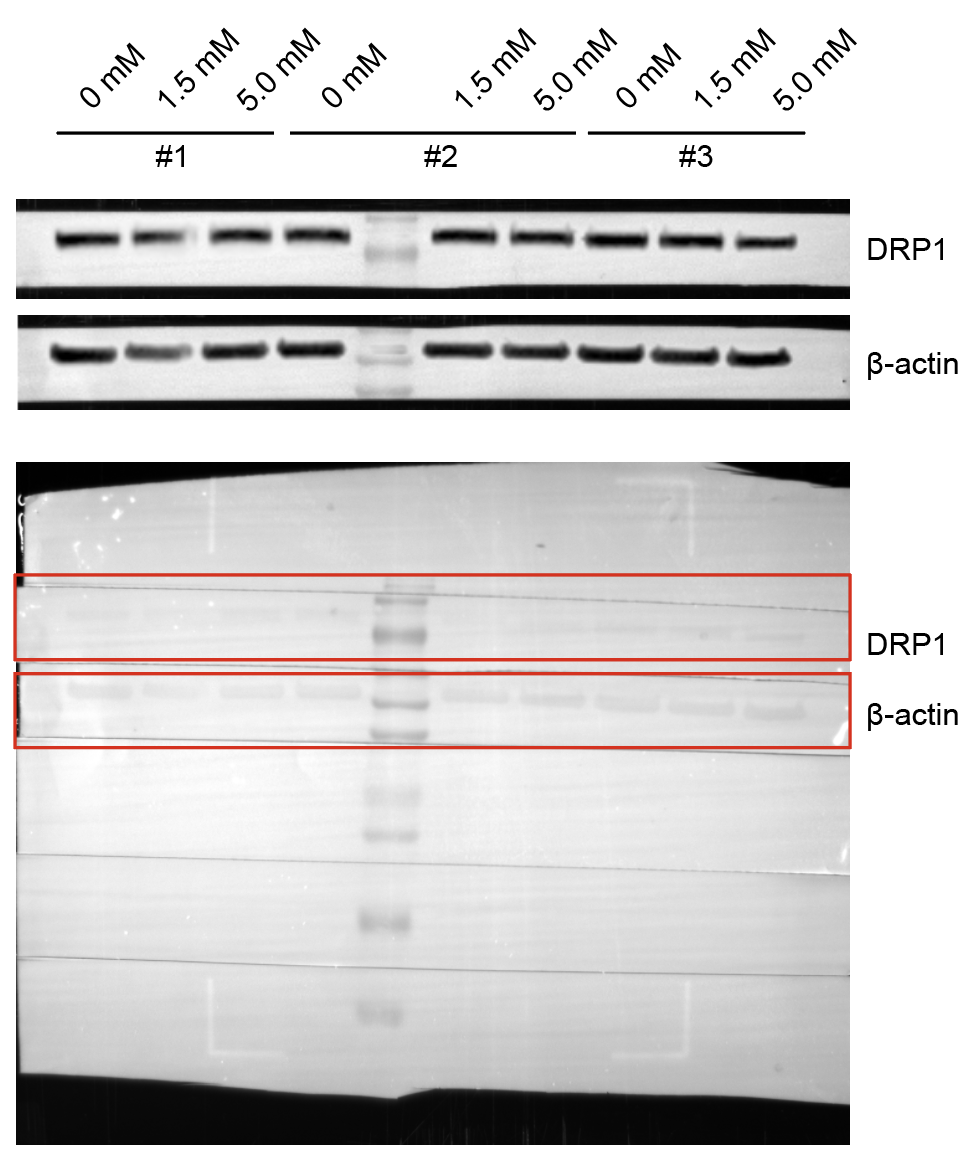
**

**
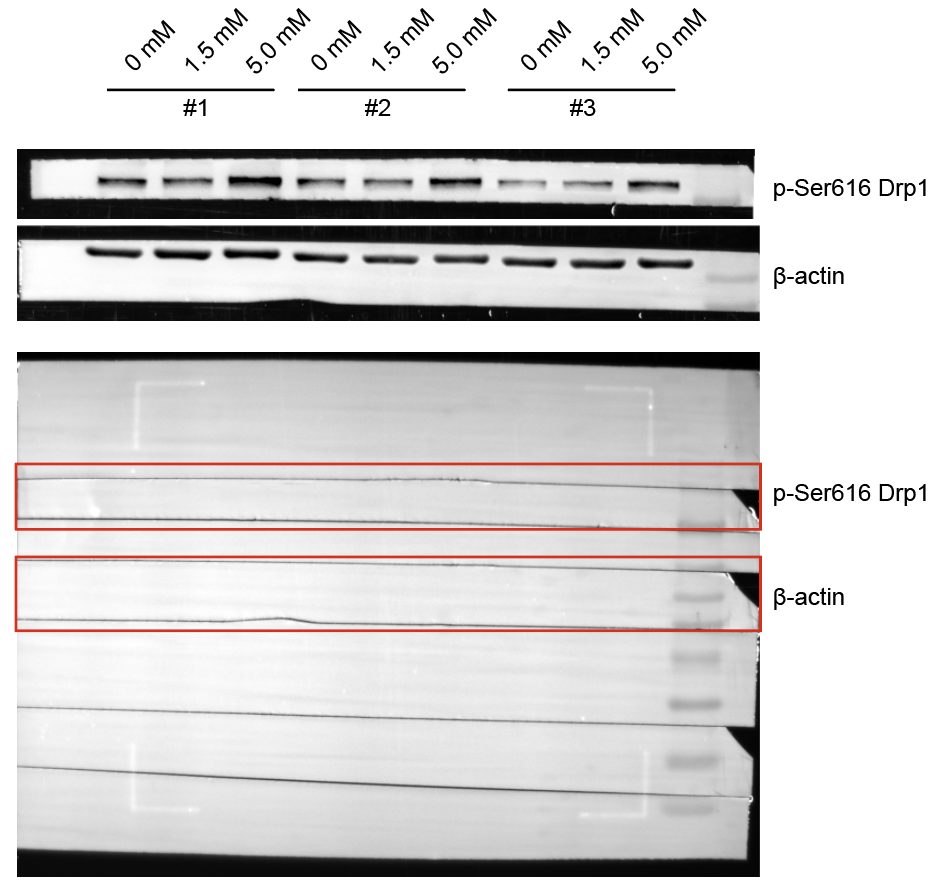
**

**
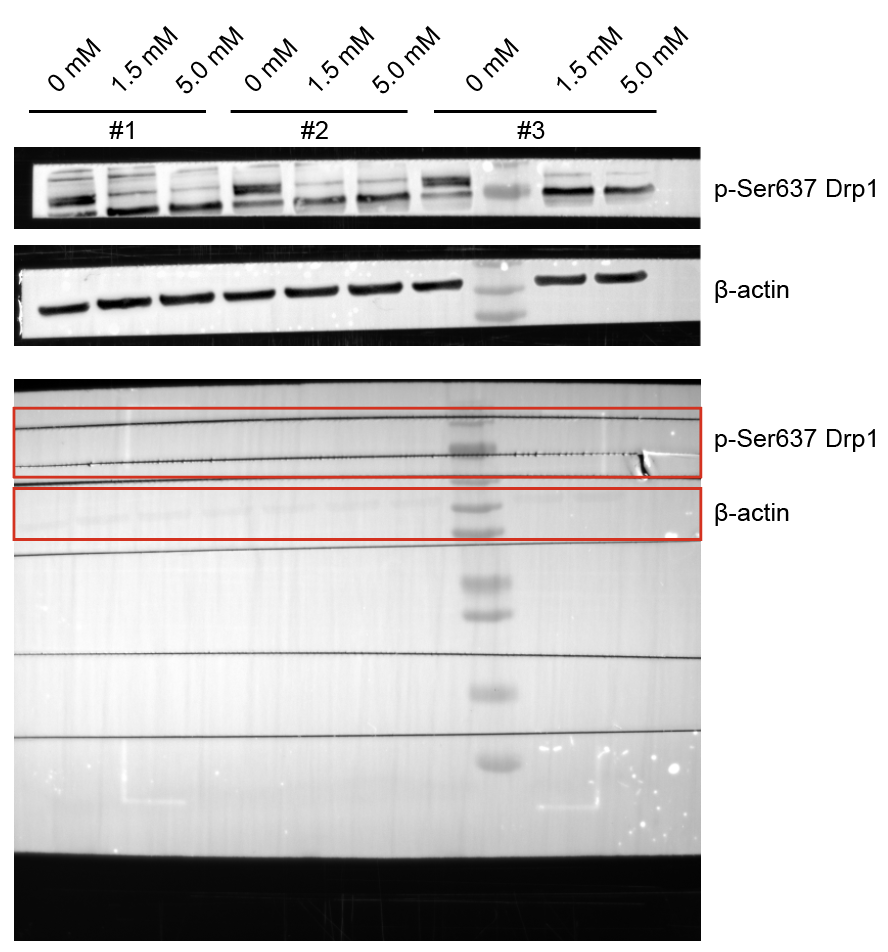
**

**Fig. 5** **Endoplasmic reticulum stress caused by iron overload in KGN cells. B** Comparison of *BIP* and *CHOP* in KGN with FAC treated and the control group (n = 3).

**
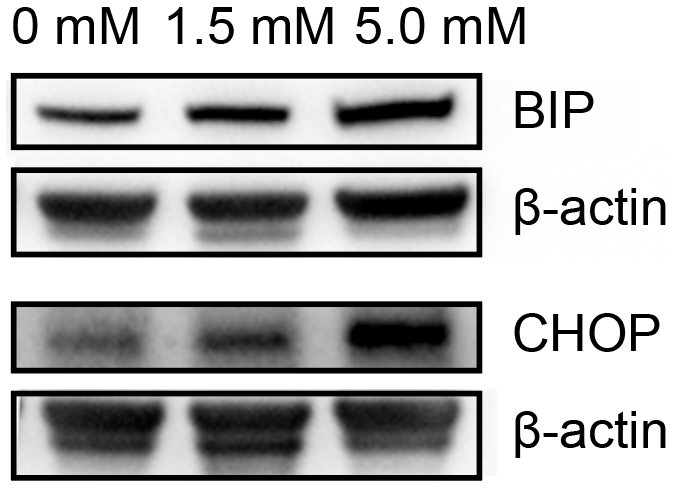
**

**Full length Western Blots:**

**
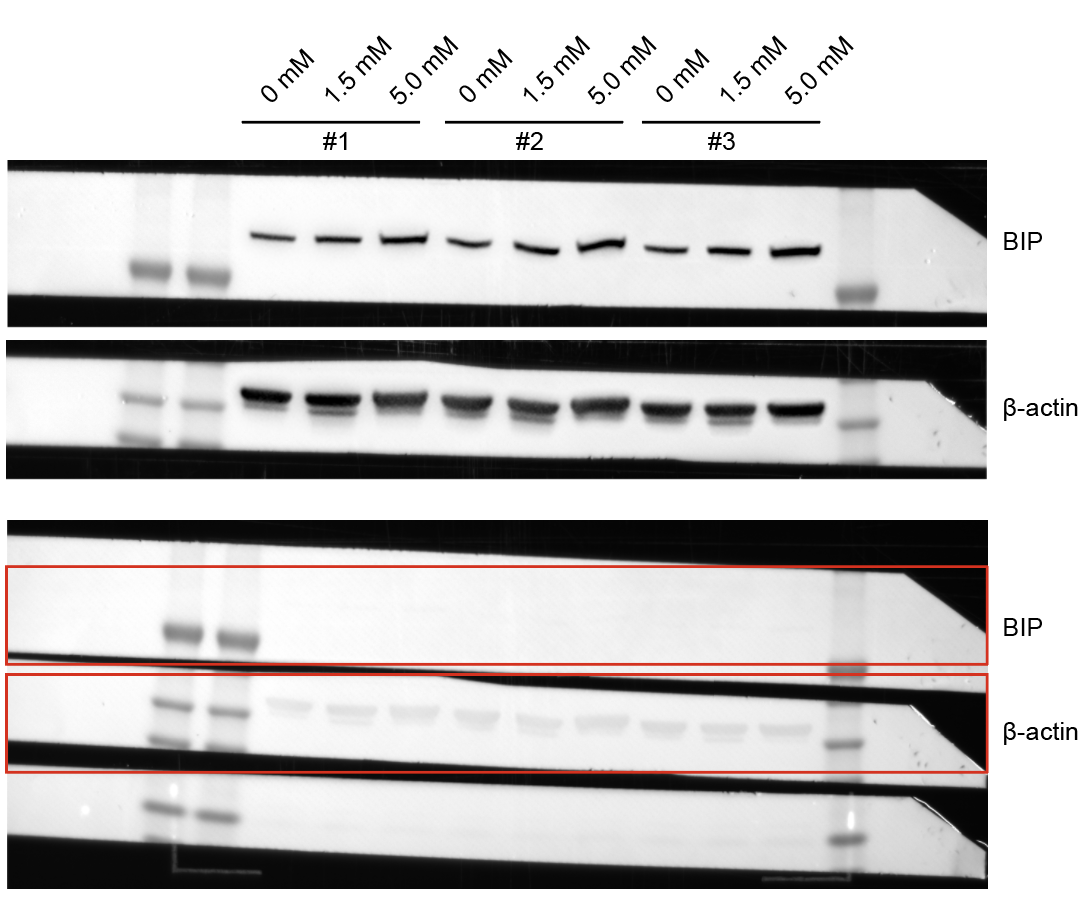
**

**
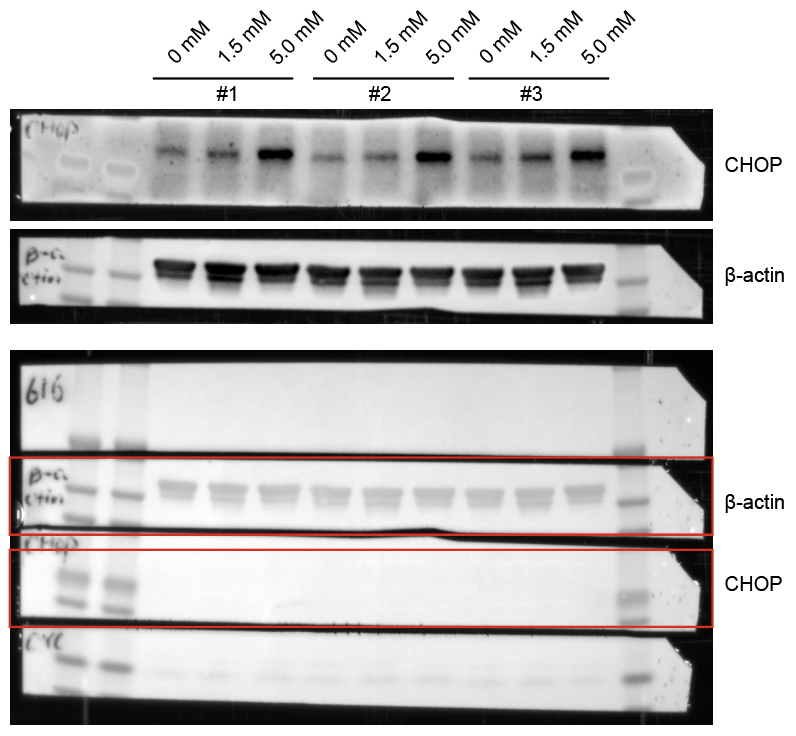
**
